# Resting-state brain information flow predicts cognitive flexibility in humans

**DOI:** 10.1038/s41598-019-40345-8

**Published:** 2019-03-07

**Authors:** Oliver Y. Chén, Hengyi Cao, Jenna M. Reinen, Tianchen Qian, Jiangtao Gou, Huy Phan, Maarten De Vos, Tyrone D. Cannon

**Affiliations:** 1 Department of Psychology, Yale University, New Haven, CT USA; 2 Department of Engineering Science, University of Oxford, Oxford, UK; 3 IBM Watson Research, New York, NY USA; 4 Department of Statistics, Harvard University, Cambridge, MA USA; 5 Department of Mathematics and Statistics, The City University of New York, New York, NY USA; 6 Department of Psychiatry, Yale University, New Haven, CT USA; 7 Fox Chase Cancer Center, Philadelphia, PA USA; 8 School of Computing, University of Kent, Canterbury, UK

## Abstract

The human brain is a dynamic system, where communication between spatially distinct areas facilitates complex cognitive functions and behaviors. How information transfers between brain regions and how it gives rise to human cognition, however, are unclear. In this article, using resting-state functional magnetic resonance imaging (fMRI) data from 783 healthy adults in the Human Connectome Project (HCP) dataset, we map the brain’s directed information flow architecture through a Granger-Geweke causality prism. We demonstrate that the information flow profiles in the general population primarily involve local exchanges within specialized functional systems, long-distance exchanges from the dorsal brain to the ventral brain, and top-down exchanges from the higher-order systems to the primary systems. Using an information flow map discovered from 550 subjects, the individual directed information flow profiles can significantly predict cognitive flexibility scores in 233 novel individuals. Our results provide evidence for directed information network architecture in the cerebral cortex, and suggest that features of the information flow configuration during rest underpin cognitive ability in humans.

## Introduction

The human brain is a dynamic system whose function is built upon communications between spatially distinct areas. Although traditional approaches using undirected functional connectivity have advanced our understanding of the functional architecture of the brain and its relationship to cognition and behavior^1,2^, the directional pattern of information flow across the brain and its relationship to human behavior are largely unknown.

Answering these questions requires another approach, so-called “effective connectivity”, which uses functional imaging data to estimate causal relationships between separated brain regions and thus quantifies information flow directions^3,4^. Previous studies using data from magnetoencephalography (MEG)^5^ have shown that brain information flow during rest is not random, but follows a posterior-to-anterior flow in high frequency bands and an anterior-to-posterior flow in low frequency bands. Data from electroencephalography (EEG)^6,7^ and functional magnetic resonance imaging (fMRI)^8,9^ have also demonstrated that the information flow in the brain is associated with the embedded network topology, such that the flow is more likely to occur from low-degree regions to high-degree regions. These results suggest that information flow in the brain possibly relates to the anatomical and functional bases of the brain architecture. Nevertheless, the exact pattern of information transfer at the regional and systems level is still unclear.

The human brain is efficiently organized to facilitate information exchange in order to support higher cognitive functions^10,11^. Consequently, the underlying information flow should subserve human cognition. Prior work indicates that executive functioning — the ability to modulate behavior to achieve a certain goal — strongly relates to the strength of information flow in the brain. For instance, effective connectivity patterns of subregions in the frontoparietal network could distinguish distinct domains of executive functions^12,13^. Research using effective connectivity approaches has also linked cognitive control to information flow in a top-down network from rostral and caudal prefrontal cortex to premotor regions^14^. Further, alterations in cortical effective connectivity have been widely reported in patients with psychiatric disorders that are characterized by executive functional deficits, such as attention-deficit hyperactivity disorder (ADHD)^15^ and obsessive compulsive disorder (OCD)^16^. These lines of evidence point to a potential link between the strength of information flow and executive ability in humans.

Using resting-state fMRI data acquired from the Human Connectome Project (HCP) in 783 healthy adults, we investigated the pattern and cognitive correlates of whole-brain information flow under a modified Granger-Geweke causality framework that accounts for subject- and edge-specific lags in brain effective connectivity. We first obtained the directionality, strength, and variability of information flow across the entire brain using a predefined atlas of 268 regions^1,17^ in a subsample of 550 healthy subjects (70% of the total sample). We then trained a regression model to identify edges correlated with measures of cognitive flexibility and employed these edges to predict cognitive flexibility scores in an independent sample of 233 healthy subjects (30% of the total sample). We hypothesized that (1) heteromodal cortical regions may show higher information flow strength and variability as well as differential information directions compared with unimodal and subcortical regions; and (2) information-flow profiles associated with higher between-subject variability are predictive of executive ability in humans.

## Results

### Individual-level whole brain information flow map

**Figure 1:**
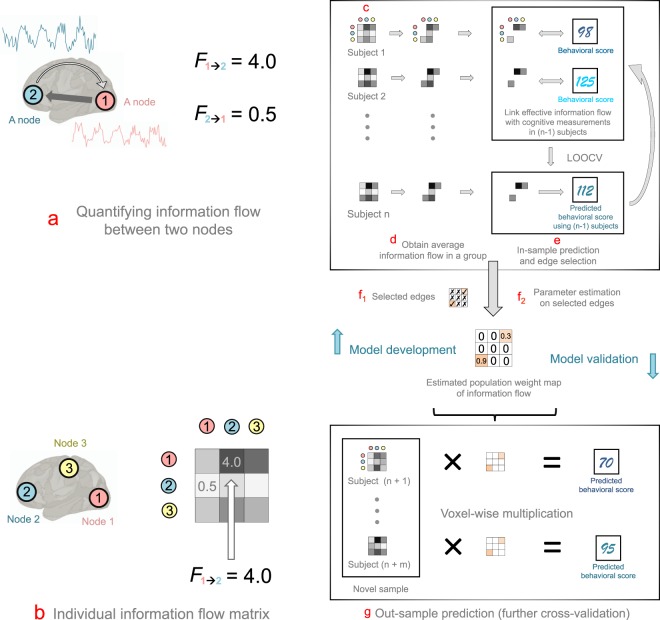
A flow chart for extracting population information map and conducting out-of-sample prediction for cognitive measurement using information flow in the human brain. (**a**) Obtaining information flow metrics (*F*-values) between every pair of regions. For time courses from brain regions 1 and 2, we obtained two directed values *F*_1→2_ (information flow from region 1 to 2) and *F*_2→1_ (information flow from region 2 to 1). (**b**) Arranging *F*-values as an information flow matrix. Specifically, *F*-values obtained from (**a**) were arranged corresponding to brain regions in an asymmetrical matrix. (**c**) Obtaining individual information flow matrix. For every subject, we computed the *F*-values for every pair of time courses. This yielded a 268 × 268 subject-specific asymmetrical *F*-value matrix and a 268 × 268 asymmetrical *p*-value matrix (corresponding to the *F*-value matrix) for each subject. (**d**) Obtaining average information flow in a group. We first computed the average *p*-values for edge across subject. We recorded edges with corresponding *F*-values that had an average *p*-value less than a threshold (*e*.*g*. 0.05). (**e**) Edge (*F*-value) selection. We used a two-step feature selection procedure. During the first step, we selected an *F*-value if its average *p*-values across subjects were below a threshold (see Methods for details). We then used a leave-one-subject-out cross-validation (LOOCV) analysis to conduct a further feature selection on *F*-values, and build a predictive model. The training and testing in LOOCV were performed iteratively for *n* times. (**f**_**1**_) Due to the large variability of LOOCV, we took the union of the selected features from each LOOCV iteration. (**f**_**2**_) Using selected *F*-values, we built a feature weight map of information flow. (**g**) Out-of-sample prediction. We multiplied the information weight map with *F*-values from previously unseen subjects, without further model fitting, to predict their cognitive scores. The efficacy and predictive power of information flow in predicting cognitive flexibility was evaluated by correlating the predicted and observed cognitive measurements.

We used eyes-open resting-state scans of 783 subjects from the HCP 1200 data release (age 22–36 years, 383 males and 400 females). The HCP 1200 data release had 1096 subjects in total. We excluded 313 subjects due to loss of time points and/or high head motion during the scans (see Methods). Each subject was scanned for two resting sessions (REST 1 and REST 2) over a period of two days. During each session, data were collected using both the left-right (LR) and right-left (RL) phase-encoding runs. For each subject, we concatenated the data (REST 1 LR, REST 1 RL, REST 2 LR, REST 2 RL) into a 3,456 seconds time-course (containing 4800 time points, with TR = 720ms). In this study, the information flow strength between two brain regions was quantified by the directed Geweke *F*-values^18^ between their time courses (see Methods). The directed Geweke *F*-value, or *F*_*i*→*j*_, is a feedback measure, which quantifies the Wiener-Granger^19–21^ causal effect of a time series *i* on another time series *j*. A large *F*_*i*→*j*_ indicates that, using past information of both *i* and *j* better predicts future values of *j*, than using only past information of *j*. This is called *i* causes *j* in the sense of Wiener-Granger causality^18^. In previous studies, the *F*-values were used to measure the “strength of causality” between two time series^18,20^. Here we extended this concept by introducing ${F}_{i\to j}^{k}(l)$ as an information flow metric in the brain. Specifically, ${F}_{i\to j}^{k}(l)$ quantified subject-specific (indexed by subject *k*) directed connectivity strength between two brain regions (denoted by *i* → *j*) with lag-length (indexed by *l*). The optimal lag *l* between two regions was determined by the Akaike information criterion (AIC)^22^, which essentially balanced the trade-off between the goodness of fit and the parsimony of the prediction model. The *F*-value varies in brain space (denoted by different *i’s* and *j’s*) and across subjects (specified by different *k’s*). A large *F*-value indicates potential strong effective connectivity between two brain regions (see Fig. 1 (a)).

Using this approach, we constructed a whole-brain information flow map, based on a functional brain atlas of 268 nodes across the whole brain^17^. For each subject, we performed Granger-causality analysis (GCA) on time courses recorded from every pair of nodes in the atlas. This yielded a 268 × 268 *asymmetrical* information flow matrix for each subject. Each entry of the information matrix represented a directed edge between two nodes. For example, the (*i*, *j*)^th^ entry of the matrix represents the strength of the information flow from node *i* to *j*; similarly, the (*j*, *i*)^th^ entry denotes the strength of the information flow from node *j* to *i* (see Fig. 1 (b)). Additionally, GCA yielded one *p*-value for every *F*-value.

**Figure 2:**
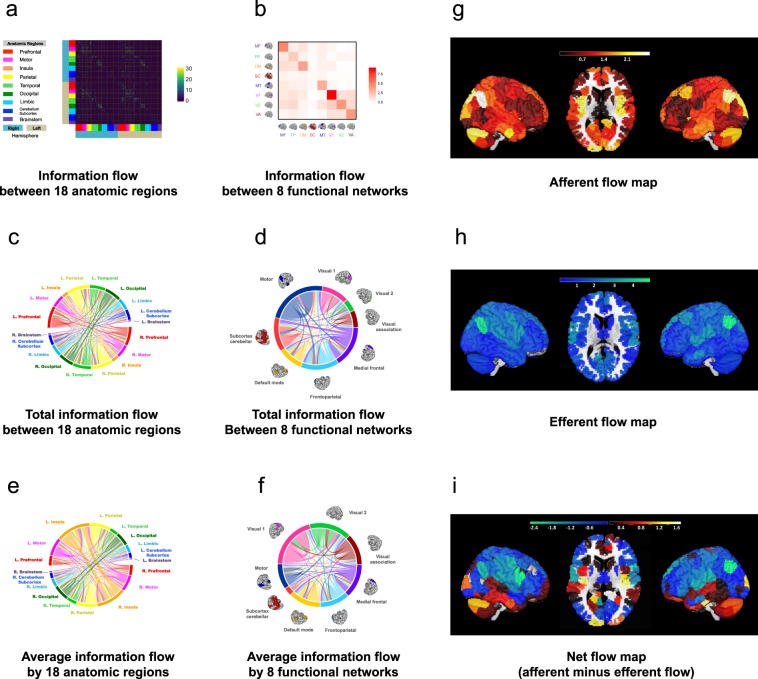
Average whole-brain information flow in a group. (**a**) The 268 × 268 *asymmetrical* information flow matrix averaged from 550 individual information matrices. The matrix is defined on 18 anatomic regions using a 268-node functional atlas. The atlas is based upon an independent data set of healthy control subjects using a group-wise spectral clustering algorithm^17^. Every entry contains a selected *F*-value from the lag-adjusted Granger-Geweke test between two time courses, or 0, if the *F*-value is not selected. An edge is selected if its average *p*-value across all subjects is smaller than 0.05. (**b**) The 8 × 8 asymmetrical information flow matrix defined on 8 functional networks. The value for each entry is the mean *F*-values associated with a functional brain network summarized from the 268 × 268 matrix in (**a**). (**c**) Total information flow map between 18 anatomic regions. The map contains 3,927 significant directed edges organized on 18 regions as in (**a**). The color of the edge indicates the origin of the flow. For example, the red curved line crossing the circle starting from the left (red bar) to the lower right (light orange bar) indicates information flow from left prefrontal region to right insula region. (**d**) Total information flow map between 8 functional networks. The same 3,927 edges in (**c**) now visualized on 8 functional brain networks. (**e**) Average information flow between 18 anatomic regions. The total amount of information flow from each anatomic region specified in (**c**) divided by the number of node in the region. (**e**) Average information flow between 8 functional networks. The total amount of information flow from each functional network specified in (**d**) divided by the number of node in the region. (**g**–**i**) Afferent, efferent, and net information flow maps of the whole brain. The brain images in (**b**, **d** and **f**) are adapted by permission from RightsLink Permissions Springer Customer Service Centre GmbH: Springer *Nature Neuroscience* “Functional connectome fingerprinting: identifying individuals using patterns of brain connectivity” by Finn *et al*. Copyright 2015.

The 268 nodes are located in a total of 18 anatomic regions; they can be further allocated into eight functional networks according to previous work (*i*.*e*. the medial prefrontal, frontoparietal, default-mode, subcortical-cerebellum, motor, primary visual (V1), secondary visual (V2), and visual association)^17^. Since different anatomic regions have various numbers of nodes, we examined both the total and the average *F*-values with regards to each region to reduce bias caused by the parcel size difference between anatomic regions (see Fig. 2).

### The magnitude of the information flow

At the regional level, we observed relatively large mean *F*-values in the prefrontal (*μ*_*F*_ = 2.18[L] and 1.77 [R]; we used L and R to denote left and right hemisphere and *μ*_*F*_ to denote the mean *F*-value in an anatomic region), motor (*μ*_*F*_ = 3.58 [L] and 2.31 [R]), temporal (*μ*_*F*_ = 2.92 [L] and 1.77 [R]), parietal (*μ*_*F*_ = 3.26 [L] and 4.04 [R]) and occipital cortex (*μ*_*F*_ = 4.67[L] and 6.59 [R]) (see Fig. 2). Compared to the global mean of *F-* values at 0.78, this suggests that there exists substantial information exchange between cerebral regions. In comparison, the brainstem had the least information exchange with the cerebral cortex (*μ*_*F*_ = 0.004[L] and 0.03 [R]) (see Fig. 2 (a, c and e)).

At the network level, our analyses demonstrated that (1) compared to the global mean of *F*-values at 0.78, there were relatively large information exchanges within each functional network (*μ*_*F*_ > 1.24), and (2) between functional networks, larger information exchange existed between the medial prefrontal (MF) and default mode (DM) (*μ*_*F*_ = 1.64 [MF → DM] and *μ*_*F*_ = 1.32[DM → MF]), between medial prefrontal and frontoparietal (FP) (*μ*_*F*_ = 1.28 [MF → FP] and *μ*_*F*_ = 1.12[FP → MF]), from medial prefrontal and frontoparietal areas to the primary visual area (V1) and secondary visual area (V2) (*μ*_*F*_ = 1.17 [MF → V1], *μ*_*F*_ = 1.46 [MF → V2], *μ*_*F*_ = 1.54 [FP → V1], and *μ*_*F*_ = 1.97 [FP → V2]), from motor (MT) to the secondary visual area (*μ*_*F*_ = 1.53 [MT → V2]) and between visual areas (*μ*_*F*_ = 2.71 [V1 → V2], *μ*_*F*_ = 2.73 [V2 → V1], *μ*_*F*_ = 1.68 [VA → V1], and *μ*_*F*_ = 2.53 [VA → V2], where VA stands for visual associate area). As for comparison, other between functional networks had mean *F*-values that were less than or close to 1 (see Fig. 2 (b, d and f)).

### The directionality of the information flow

We further examined the directionality of information flow between different nodes, regions, and networks, and statistically compared the strength of the information flow from different directions. We found that the strongest afferent flow was primarily present in the posterior and ventral parts of the brain, including the occipital cortex, temporal cortex, insula, and cerebellum. In contrast, the afferent flow was relatively small in the lateral prefrontal cortex, sensorimotor area, and posterior temporal region. Large efferent flow was observed in the dorsal and lateral parts of the brain including the frontal cortex, sensorimotor cortex, and the parietal cortex, while relatively small efferent flow was shown in the cerebellum, subcortex, and temporal cortex (see Fig. 2 (g–i)).

Since each node is associated with both afferent and efferent flows, we further subtracted the efferent flow *F*-values from the afferent flow *F*-values for each node to calculate the “net” information flow across the whole brain. We found that the ventral part of the brain (lateral and medial temporal cortex, insula, and cerebellum) was predominantly associated with net afferent flow, while the dorsal part of the brain (mostly frontal and parietal cortices) showed net efferent flow. These results suggest that the directionality of net information flow is differentially distributed across the whole brain, and the distinct information flow patterns between the dorsal and ventral brain may be attributed to their functionality differences (see Fig. 2 (g–i)).

**Figure 3:**
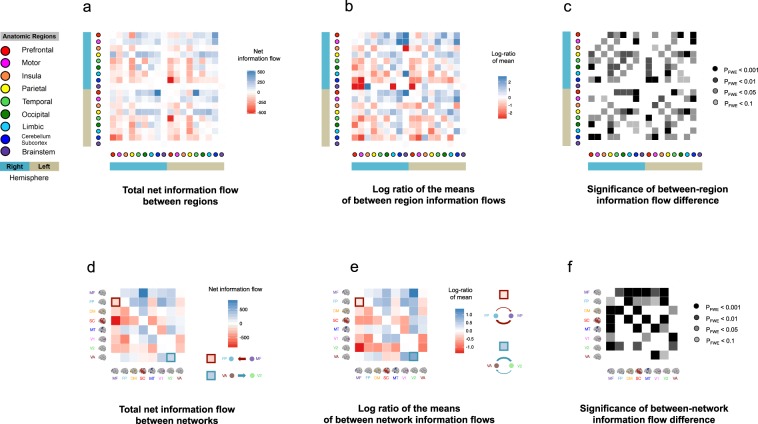
Net total information flows of the whole-brain and mean information flow difference between regions and networks. (**a**) The net total information flows of the whole brain organized by regions. Each square represents the net total amount of information flows from one brain area to the other. The darker a blue square was, then the more (net) information was flowing out from the brain region denoted by the reference brain image on the horizontal direction into the brain area denoted by the reference brain image on the vertical direction. The darker a red square was, the more (net) information was flowing into the brain region denoted by the reference brain image on the horizontal direction from the region denoted by the reference brain image on the vertical direction. (**b**) The mean difference between region information flows. Each square represents the difference between the mean of information flows from the brain area denoted by the reference brain image on the horizontal direction to the brain area denoted by the reference brain image on the vertical direction. Color conventions as in (**a**). (**c**) The *p*-values for the corresponding mean difference in (**b**), corrected by FWER. (**d**) The net total information flows of the whole brain organized by networks. Color conventions as in (**a**). (**e**) The mean difference between network information flows. Color conventions as in (**a**). (**f**) The *p*-values for the corresponding mean difference in (**e**), corrected by FWER. The brain images in (**d**, **e** and **f**) are adapted by permission from RightsLink Permissions Springer Customer Service Centre GmbH: Springer *Nature Neuroscience* “Functional connectome fingerprinting: identifying individuals using patterns of brain connectivity” by Finn *et al*. Copyright 2015.

Next, we examined the directionality of information flow in the 18 anatomical regions (see Fig. 3 (a,b)). The results revealed that the motor (*μ*_*F*_ = 4.42 [L], *μ*_*F*_ = 4.46[R]), parietal (*μ*_*F*_ = 4.74 [L], *μ*_*F*_ = 4.95 [R]), and prefrontal (*μ*_*F*_ = 4.00 [L], *μ*_*F*_ = 3.88 [R]) regions were primarily associated with net efferent information, whereas the insula (*μ*_*F*_ = 4.63 [L], *μ*_*F*_ = 4.26 [R]), limbic (*μ*_*F*_ = 3.27 [L], *μ*_*F*_ = 3.52 [R]), brainstem (*μ*_*F*_ = 2.45 [L], *μ*_*F*_ = 2.45 [R]), cerebellar and subcortical (*μ*_*F*_ = 3.48 [L], *μ*_*F*_ = 3.47 [R]) regions were primarily associated with net afferent flow. The temporal and visual regions were associated with a more balanced information inflow and outflow. These findings suggest that the overall directionality of resting-state information flow is from the dorsal brain to the ventral brain.

The network-level results revealed that the medial frontal (*μ*_*F*_ = 4.56), frontoparietal (*μ*_*F*_ = 4.17), and visual association (*μ*_*F*_ = 3.87) subnetworks were primarily associated with net efferent flow, while the secondary visual (V2) (*μ*_*F*_ = 4.20) and subcortico-cerebellar (*μ*_*F*_ = 3.00) subnetworks were primarily associated with net afferent information. The other subnetworks (default-mode, motor, and primary visual) were associated with a more balanced information inflow and outflow (see Fig. 3 (d and e)). These findings suggest substantial information flow from high-order cognitive systems to primary functional systems during resting state.

Finally, to investigate whether the net information flow was statistically significant, we conducted a pair-wise ANOVA (analysis of variance) study of the net information flows (specifically, we performed ANOVA for the *F* values associated with each pair of brain regions). We tested if there was a difference between information flow from area A to area B and that from area B to area A, for each pair of networks (see Fig. 3 (c and f)). Our analyses showed that there was significant net information flow associated with medial frontal, default mode, and visual networks (P_FWE_ < 0.001, where P_FWE_ denotes adjusted *p*-value controlling for family wise error rate (FWER)). Taken together with the directionality of the net information flow from Fig. 3 (b), these results suggest that the medial frontal and frontoparietal subnetworks were associated with significant net efferent flow, and the visual association subnetwork was associated with significant net afferent information flow.

### Analysis of variability of the whole-brain information flows

In humans, the brain is the most variable and fastest evolving organ^23^. Within the brain, different regions may be associated with different variabilities in information flow. The characteristics of the variability associated with information communication across different brain networks, however, are not well-charted. To inquire into this property, we performed an analysis to investigate the variability of the information flow.

To quantify between-subject variability, we first calculated the variance of *F*-values (directed edges) across 550 subjects in the training sample. We found that edges with relatively high afferent flow variance generally involved those from the whole brain to the parietal (*Ratio* = 1.34 [L] and 1.65 [R], both P_FWE_’s < 0.001, where *Ratio* refers to the ratio between the average variability of the afferent information flow of a particular region and that of the whole brain, and [L] and [R] refer to the left and right hemispheres, respectively), from the whole brain to insula (*Ratio* = 1.43 [L] and 1.22 [R], both P_FWE_’s < 0.01), and from the whole brain to occipital (*Ratio* = 1.35 [L] and 1.47 [R], both P_FWE_’s < 0.001) regions, suggesting high between-subject variability of information flow. In contrast, edges within the cerebellar-subcortical (*Ratio* = 0.86 [L] and 0.86 [R], both P_FWE_’s < 0.001), brain stem (*Ratio* = 0.39 [L] and 0.37 [R], both P_FWE_’s < 0.001), and limbic (*Ratio* = 0.75 [L] and 0.90 [R], both P_FWE_’s < 0.001) regions showed relatively low variance, suggesting that afferent flows in these regions are relatively stable across subjects (see Fig. 3).

Edges with relatively high efferent flow variance generally involved those from the PFC region to the whole brain (*Ratio* = 1.25 [L] and 1.16 [R], both P_FWE_’s < 0.001), from the motor region to the whole brain (*Ratio* = 1.35 [L] and 1.37 [R], both P_FWE_’s < 0.001), from the parietal region to the whole brain (*Ratio* = 1.62 [L] and 1.88 [R], both P_FWE_’s < 0.001), and from the occipital to the whole brain (*Ratio* = 1.35 [L] and 1.24 [R], both P_FWE_’s < 0.001), suggesting high between-subject variability of information flow. In contrast, edges within the cerebellar-subcortical (*Ratio* = 0.69 [L] and 0.74 [R], both P_FWE_’s < 0.001), brain stem (*Ratio* = 0.28 [L] and 0.25 [R], both P_FWE_’s < 0.001), and limbic (*Ratio* = 0.56 [L] and 0.60 [R], both P_FWE_’s < 0.001) regions showed relatively low variance, suggesting that efferent flows in these regions are relatively stable across subjects (see Fig. 3).

**Figure 4:**
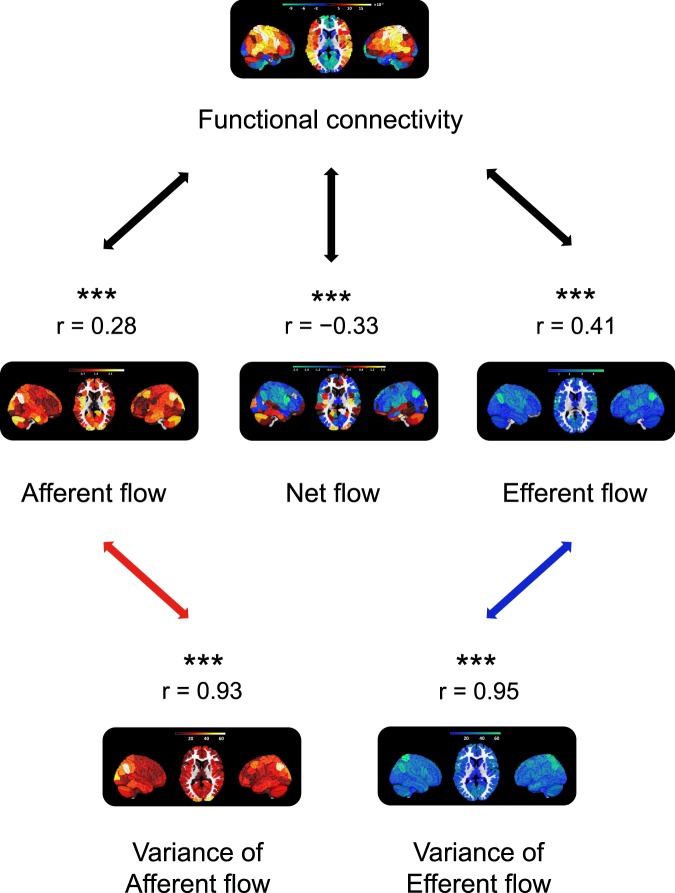
A comparison between functional and effective connectivity. The functional connectivity map was obtained by first averaging 550 268 × 268 Pearson correlation matrices obtained from 550 subjects. Subsequently, the resulting 268 × 268 matrix was further summarized according to 268 brain areas. This yielded a vector containing 268 values, each of which corresponds to the “connectiveness” of a brain area to the rest of brain areas. The effective connectivity map was obtained by first averaging 550 268 × 268 *F*-matrices obtained from 550 subjects. Subsequently, the resulting 268 × 268 matrix was further summarized according to 268 brain areas, into three vectors. The first vector contained 268 values, each of which corresponds to how much information flow is flowing into a particular brain area from other brain areas (afferent flow). The second vector also contained 268 values, each of which corresponds to how much information flow was flowing out from a particular brain area to other brain areas (efferent flow). The third vector was obtained by subtracting the efferent flow from the afferent flow, indicating the net information flow entering each brain area. Finally, the association between functional and effective connectivities can be quantified by correlating the functional connectivity vector with each of the three effective connectivity vectors.

Interestingly, we observed high correlations between the variability maps and the information flow strength (*F*-value) maps (Fig. 4), suggesting that regions with high information flow strength also had high information flow variability, and *vice versa*.

Next, we compared the variability of the information flow obtained from the eight functional networks. We began by taking the ratio of the variability within each network and that of the whole brain. A positive ratio indicated that the within network variability was greater than that of the whole brain, and a negative ratio indicated that the within network variability was smaller than that of the whole brain. To quantitatively demonstrate whether the above differences were significant, we performed an ANOVA test between the variability within each network and that of the whole brain. As Fig. 4 (d) shows, most networks had significantly higher variability (P_FWE_ < 0.001) than the whole brain, except the subcortical cortex.

**Figure 5:**
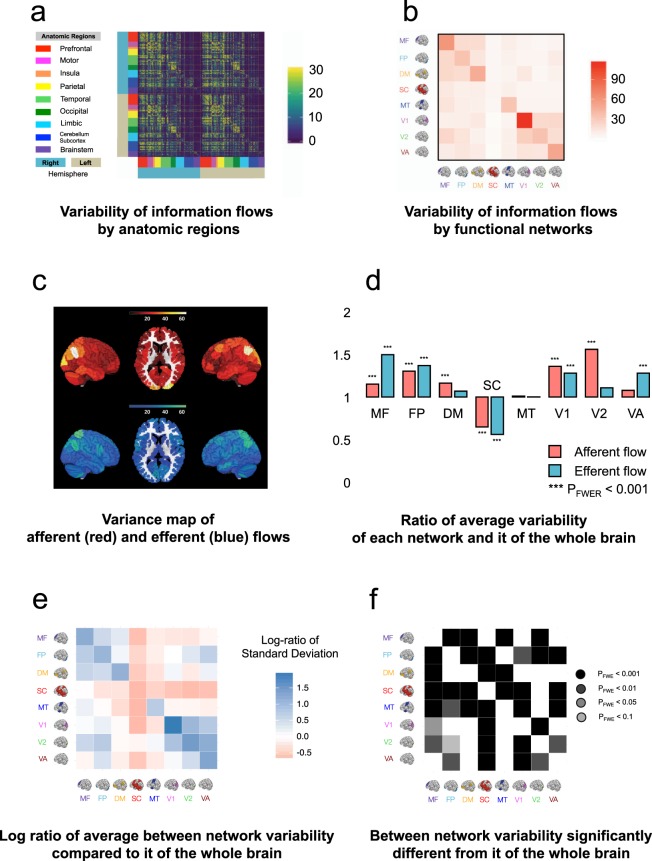
Analysis of variance of the whole-brain information flows. (**a**) The variance of 268 × 268 asymmetrical information flow map across 550 subjects. Every entry contains the variance of the *F*-value between two time courses across subjects. In the figure, for color contrast convenience, if an *F*-value is greater than 100, we fix it at 100. (**b**) The variance of 8 × 8 asymmetrical information flow map across all subjects defined on 8 functional networks. Every entry is the mean variance of *F*-value associated with a functional brain network summarized from the 268 × 268 matrix in (**a**). (**c**) The variability map of afferent (in red color) and efferent flows (in blue color). (**d**) The variability of afferent (red) and efferent flows (blue) associated with each network compared to it of the whole brain. The height of each histogram quantifies the average variance of *F*-values corresponds to each network, which were visualized in panel (c), compared to it of the whole brain. A pair-wise ANOVA test determines the significance level. All *p*-values were adjusted for FWER. (**e**) The log-ratio of average variation of information flows between each pair of networks. Each log-ratio was calculated in two steps. First, we found the average variations of the efferent flow from nodes linking regions A and B - it measures the average variability of the efferent flow from region A to region B. We also found the average variations of the afferent flow from regions B to A - it measures the average variability of the afferent flow from region A to region B. Second, we calculated the natural log of these two average variations. The darker a blue square was, the more variability of information flowing out from the brain region denoted by the reference brain image on the horizontal direction than the variability of the opposite information flows; the darker a red square was, the more variability of information flowing into the brain region denoted by the reference brain image on the horizontal direction than the variability of the opposite information flows. (**f**) The significance of the between-network variability. The darker the green square, there was more significant a difference between the variability of the information flows from opposite directions (namely, the variability of information flow from region A to B compared to it from region B to A). The size of the difference (namely, whether there is more variability of the information flow from region A to B than it from B to A) can be determined from the map in (**e**). The comparison was done using a pair-wise ANOVA test. All *p*-values were adjusted for FWER. The brain images in (**e**) and (**f**) are adapted by permission from RightsLink Permissions Springer Customer Service Centre GmbH: Springer *Nature Neuroscience* “Functional connectome fingerprinting: identifying individuals using patterns of brain connectivity” by Finn *et al*.^1^.

Finally, we analyzed the variability of between-network information flows. To that end, we computed the log ratios of the variability between the afferent and efferent flows in each pair of networks. Specifically, for each two networks A and B, we calculated the natural logs of the average variance from A to B and that from B to A (see Fig. 5 (e)). The derived log values were further divided by each other to generate a ratio. Next, we conducted a pair-wise ANOVA test to compare the log ratios of every pair of networks with that of the whole brain to examine whether the differences between variability of information inflow and outflow were statistically significant. Our results showed that, compared to the variability of information flow in the whole brain, there was significantly higher information flow variability among medial frontal cortex, frontal parietal regions, the default mode regions (all P_FWE_’s between each two networks < 0.001), suggesting that information flow between association cortices is highly variable. Additionally, there was significantly higher information flow variability among different visual networks (all P_FWE_’s between each two networks < 0.001, see Fig. 5 (f)).

As a whole, our analyses both qualitatively and quantitatively suggest that there is substantial variability of information flow within each within brain networks compared to the whole brain. Between-network information flow is associated with higher variability between higher-order cognitive systems (*i*.*e*. medial prefrontal, frontoparietal, and the default-mode) and between different visual areas.

### Prediction of cognitive flexibility

Inspired by prior work, which showed that information flow in the brain was related to executive functioning^13,14^, we further investigated whether directed information flow among those edges selected during the first step edge selection could predict individual executive ability in the healthy population. The executive ability was evaluated by the Dimensional Change Card Sort (DCCS) Test^24^ in the HCP data. The DCCS test is a widely used neurocognitive task that assesses individuals’ cognitive flexibility by rapidly switching card-sorting rules between different dimensions. A higher performance score reflects greater flexibility in monitoring and switching thought and behavior to facilitate the attainment of target goals.

The behavior prediction procedure can be, broadly, summarized in three steps. First, we conducted the first step edge selection to remove spurious connections possibly caused by noise. During the first step we selected an edge, if its average *p-value* (obtained from the GCA) across all subjects was smaller than a threshold (*e*.*g*. *p* < 0.1) (see Remarks in the supplementary materials). Next, we performed a second step edge selection and model building. We followed prior work^1^ and used a machine-learning-based framework with leave-one-subject-out cross-validation (LOOCV) on a randomly selected subsample of 550 healthy subjects (70% of the total samples). The selected edges were employed to fit a linear regression model, where edge strengths (*F*-values) and covariates (*i*.*e*. age and gender) were entered as regressors. Edges that were significantly correlated with the DCCS scores at *p* < 0.005^25,26^ were identified as “effective information flows”, and were selected as neural biomarkers. The identified effective flows were subsequently isolated into positive and negative groups, based on the sign of their correlations with DCCS scores. During each LOOCV, we applied the estimated regression weights using data from 549 training subjects to data from the holdout, and obtained a predicted DCCS test score. We iterated the LOOCV analysis 550 times, where each subject’s DCCS score in the sample was predicted once using other subjects’ data. We built the predictive edges into an *F*-value weight map (as the weights from a regression analysis), where each entry of the map indicated the weight of a predictive *F*-value (see Fig. 1(f)).

To investigate the robustness and reproducibility of the results, we extended the prediction analysis to the held-out sample of 233 subjects (testing set, or 30% of the total sample). *F*-values for each novel subject in the testing set were extracted and then multiplied by the weight map obtained from the training set, yielding a predicted DCCS test score for each subject in the testing set (see Fig. 1). To evaluate prediction power, we correlated all predicted and observed DCCS scores of subjects from the training dataset using Pearson correlation. One thousand nonparametric permutation tests were performed to evaluate model fitting and out-of-sample prediction. During each test, we randomly permuted the DCCS scores, and then conducted model fitting using the same procedure as described above. Prediction power for each permutation test was recalculated.

**Figure 6:**
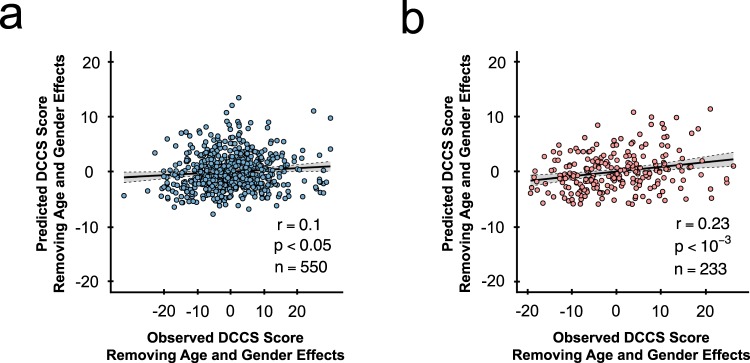
Individual effective information flow predicts cognitive flexibility. (**a**) LOOCV prediction result comparing predicted and observed DCCS scores (*n* = 550 subjects). Confounds, such as age and gender, are regressed out before prediction. Scatter plot shows predication based upon the whole brain positive effective information flows (threshold at *p* < 0.005). Each dot represents one subject; grey area indicates the 95% confidence band for best-fit line. (**b**) Out-of-sample prediction result comparing predicted and observed adjusted DCCS scores (*n* = 233 subjects). Confounds, such as age and gender, are regressed out before prediction. Scatter plot shows predication based upon the whole brain positive effective information flows (threshold at *p* < 0.005). Each dot represents one subject; grey area indicates the 95% confidence band for best-fit line.

Our analysis revealed that effective information flow (extracted *F*-values) positively correlated with the DCCS scores in the training sample significantly predicted cognitive flexibility in the test sample. In particular, at the threshold of *p* < 0.005, 178 edges were found to be positively predictive of DCCS score. The LOOCV in the training sample showed a prediction accuracy of *r* = 0.1 (*n* = 550, *p* < 0.05) (see Fig. 6 (a)). The correlation between predicted and true values in the out-of-sample prediction was *r* = 0.23 (*n* = 233, *p* < 0.001) (see Fig. 6 (b)). Notably, the observed Pearson correlation *r* was larger than any permuted *r*-values in the permutation distribution (*p* < 0.001), suggesting a highly significant predictability than one would expect by chance. In contrast, we did not observe significant predictive effect on edges with negative correlations with the DCCS scores at any of the above thresholds (*p *> 0.05).

Because the choices of statistical thresholds during the two-step edge selection were somewhat arbitrary, a range of thresholds were tested to ensure that results were consistent. In an additional analysis, we maintained the second step edge selection threshold at 0.005 and changed the first step threshold from 0.1 to 0.05. In the training sample, the prediction is *r* = 0.11 (*p* = 0.01, *n* = 550) and out-sample prediction is *r* = 0.20 (*p* = 0.002, *n* = 233). The more conservative threshold selected fewer edges (68, as compared to 178 using the more moderate threshold). In another analysis, we maintained the first step edge selection threshold at 0.1 and considered the second step edge selection threshold at 0.01. In the training sample, the prediction is *r* = 0.07 (*p* = 0.1, *n* = 550) and out-sample prediction is *r* = 0.21 (*p* = 0.001, *n* = 233). However, the increasing threshold selected significantly more edges (303, as compared to 178 using the more conservative threshold).

**Figure 7:**
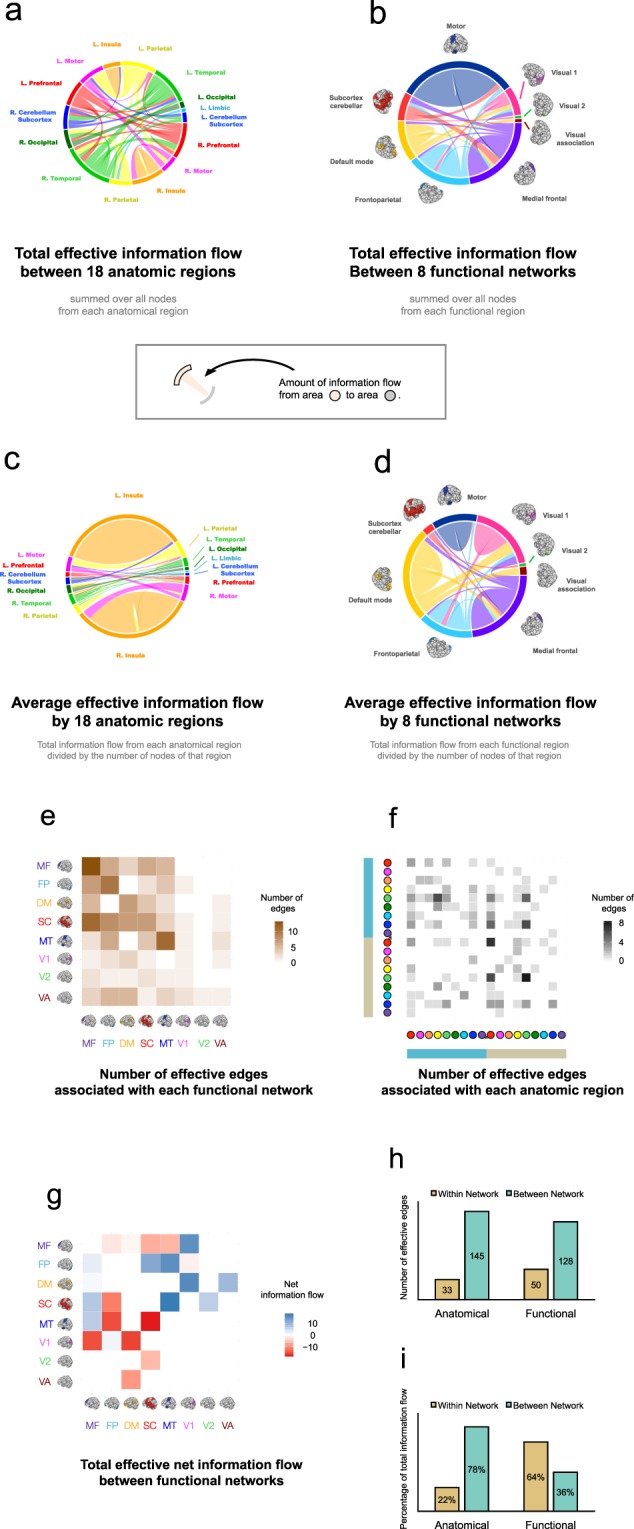
Individual effective information flows. (**a**) The information map of positive total effective information flow edges based upon feature selection. The flows are arranged according to 18 brain regions. (**b**) The same edges in (**a**) now organized according to 8 functional brain networks. (**c**) Average effective information flow (total effective information flow in figure (**a**) divided by the number of nodes in each anatomic region). (**d**) Average effective information flow (total effective information flow in figure (**b**) divided by the number of nodes in each functional network). (**e**) Number of effective edges arranged according to 8 functional brain regions. (**f**) Number of effective edges arranged according to 18 anatomic brain regions. Color convention as in Fig. 3 (a). (**g**) Net total effective information flow between 8 functional brain regions. The darker the black square was, then the more information is flowing out from the brain region denoted by the reference brain image on the horizontal direction to the brain region denoted by the reference brain image on the vertical direction; the darker the red was, then the more information is flowing into the brain region denoted by the reference brain image on the horizontal direction from the brain region denoted by the reference brain image on the vertical direction. (**h**) The number of effective edges arranged by within and between anatomic regions, as well as it arranged by within and between functional networks. (**i**) The total information flow (summed *F*-values) arranged by within and between anatomic regions, as well as it arranged by within and between functional networks. The brain images are adapted by permission from RightsLink Permissions Springer Customer Service Centre GmbH: Springer *Nature Neuroscience* “Functional connectome fingerprinting: identifying individuals using patterns of brain connectivity” by Finn *et al*.^1^.

Next, we aimed to unveil the details of the extracted directed edges predictive of DCCS scores. Our results showed that they were primarily present within the higher-order cognitive systems (*i*.*e*. medial prefrontal (*μ*_*F*_ = 13.28), frontoparietal (*μ*_*F*_ = 18.98) and the default-mode (*μ*_*F*_ = 12.41)), and from higher-order systems to the primary systems (*e*.*g*. frontoparietal to motor (*μ*_*F*_ = 6.09), frontoparietal to V1 (*μ*_*F*_ = 9.14), medial prefrontal to V1 (*μ*_*F*_ = 8.60), and frontoparietal to visual association (*μ*_*F*_ = 11.85)) (Fig. 7). In addition, information flow was also shown from the motor network to the medial prefrontal (*μ*_*F*_ = 8.41) and frontoparietal networks (*μ*_*F*_ = 7.51), and from the subcortical network to the medial prefrontal network (*μ*_*F*_ = 9.82). Taken together, these results suggest that information flow associated with the prefrontal cortex is critical to, and predictive of, human cognitive flexibility.

### Comparison between functional connectivity and effective connectivity

An interesting question would be whether the effective connectivity patterns examined here can be largely explained by undirected functional connectivity. If so, the effective connectivity measures may have limited practical value. For this purpose, we conducted a further analysis comparing the patterns the functional connectivity with that of the afferent flow (*i*.*e*. information flow towards each brain area), the efferent flow (*i*.*e*. information flow outwards each brain area), and the net flow (*i*.*e*. afferent flow minus efferent flow). Our analysis showed that the effective connectivity maps were only moderately correlated with the functional connectivity map (*r* = 0.28, 0.41, and −0.33 for afferent, efferent and net flow, respectively). As a result, functional connectivity can only explain between 0.08 and 0.17 (*r*^2^) of total variance in the effective connectivity matrices. These findings suggest the uniqueness of the results derived from GCA analysis, which cannot be fully accounted by functional connectivity analysis.

Furthermore, we compared the behavior prediction using effective information flow (*F*-values) and the traditional functional connectivity^1^. Using functional connectivity (*i*.*e*. Pearson correlation between times courses from different brain areas), we obtained a prediction of *r* = 0.1 in the training set (*p* = 0.001, *n* = 550) and *r* = 0.22 in the test set (*p* < 0.001, *n* = 233). This was similar to our “effective” edge-based prediction performance. This prediction results, however, were based on 342% more edges (787 functional edges as compared to 178 “effective” edges identified using information flow), suggesting that many of the correlation-based edges did not further improve behavior prediction.

## Discussion

In this study, we employed Granger-Geweke causality framework on fMRI data to study resting-state idiosyncratic whole-brain information flows in healthy individuals. The analyses revealed that information flows were mainly distributed within each specialized functional system, from the dorsal brain to the ventral brain, and from the higher-order cognitive systems to the primary functional systems. The regions with high information flow strength also showed high between-subject information flow variability. In addition, we showed that the strength of information flow between the higher-order cognitive systems and from these systems to the primary systems (visual, motor, and subcortical) predict cognitive flexibility scores in humans.

Substantial evidence has demonstrated that within-system functional connectivity is particularly strong in the brain, as compared to between-system connectivity^27,28^. This finding is consistent across different brain states^29–31^, data processing methods^32,33^ and populations^34,35^, suggesting a robust network organizational feature of the brain. In this article, we extended previous findings by showing strong effective connectivity within each functional system. Our result suggests that system-level information flow is strong between regions that are involved in the same function. In contrast, between-system effective connectivity was much weaker than within-system connectivity. Strong between-system connectivity, however, was mainly observed in the higher-order cognitive systems and from these systems to more primary functional systems. This agrees with prior findings that brain hubs are primarily distributed in the frontoparietal and the default-mode networks^36,37^, which have the most connections with other parts of the brain. Since the top-down projections from the prefrontal and higher-order systems to the primary functional systems are critical to control and coordinate human behavior^38–41^, the abundant information flow at such direction during resting state may suggest a cognitive effort to focus the attention and to predict and adapt one’s mind and behavior during the scan.

Interestingly, directed edges with the highest *F-*values also showed the largest information flow variability across subjects. The variability was particularly prominent in the frontal cortex, parietal cortex, and the visual cortex. Much evidence has shown that connectivity in the frontoparietal system is associated with low within-subject variability but high between-subject variability, which may act as “fingerprints” to distinguish individuals^1,42,43^. Further, connectivity in the cognitive control systems shows the highest vulnerability to subject-specific psychological and physiological factors, such as mood, stress, fatigue, personal experience, among others^44–47^. These findings support our results and suggest that the high variability of information flow in the frontal and parietal cortices across subjects may serve as a neural signature to discern and predict individual behaviors. Apart from these regions, high variability was also observed in the visual systems, which may relate to the continuous sensation of environmental change during the eyes-open resting state scan. This finding, however, is different from previous findings that the primary functional systems are associated with relatively small between-subject variability^48^. Such discrepancy may to some degree suggest a higher sensitivity of effective connectivity in assessing individual differences in the primary functional systems.

The information flow that predicts cognitive flexibility involved connectivity within the higher-order systems (frontoparietal, medial prefrontal, and the default-mode) and from these systems to the primary sensory-motor systems. This reflects the key role of higher-order systems in human cognition. The frontoparietal system is a crucial system for cognitive control in humans – a set of cognitive processes that coordinate and monitor goal-directed behaviors^49,50^, and the medial frontal system participates cognitive control processing mainly by evaluating the value of input information^51,52^, error detection^51,52^, conflict monitoring^53^, and decision making^54^. Previous research has revealed that functional connectivity in the frontoparietal system has the highest predictability for fluid intelligence in humans^1^. In line with prior results, we further show the highest predictability of information flow in higher-order cognitive systems for human cognitive flexibility – a critical feature of cognitive control ability. These findings together suggest that both functional and effective connectivity of higher-order systems are critical to cognitive functioning in humans.

We would like to note several limitations of our study. First, the sampling rate for fMRI data is slower than the timescale of the underlying neuronal responses. This is a common limitation for GCA studies using fMRI data^55,56^. In this work, we sought to attenuate the effect of this limitation by using the HCP data with relatively high temporal resolution (TR = 720 ms). This potentially reduces the biases caused by the mismatch of timescales. We acknowledge, however, that the findings might still be affected by this factor to a certain degree. Future work replicating these results using other data acquisition techniques, such as EEG and MEG, is important. Second, the information flow profiles and the prediction power were calculated based on the integration of large time series acquired from two days lasting for a total of one hour. The findings reported in the current study, therefore, are likely to reflect brain information configuration over a long period of time. Given the dynamic nature of connectivity measures^57,58^, information flows may dynamically change over time during the whole scan. Third, our study using GCA only captures linear information flows in the brain. Future work using non-linear parametric approaches may be useful to uncover the non-linear information flow architecture of the brain. Fourth, our results are derived from a sample of young healthy adults. Whether these findings would generalize to other populations is an open question and needs to be investigated in future studies. Fifth, although BOLD signals possess a strong neural basis as they are highly correlated with local field potential, which is a direct measure of synaptic activity^59^, we acknowledge that our results may to certain degree be influenced by vasculature.

In conclusion, our study provides evidence for the directionality and patterns of information flow in the human brain and highlights the importance of information flow in the high-order systems in relation to cognitive flexibility. Since these information flow patterns cannot be fully explained by traditional functional connectivity measures, the data presented here extend current knowledge of human brain functional organization and open a new avenue towards investigating the neurobiological basis for individual executive functional ability. These findings may also show potential to help advance our understanding of brain disorders characterized by deficits in executive functioning, such as schizophrenia, ADHD, and OCD.

## Methods

### Subject information

The data set used in this article was from the Human Connectome Project (HCP) 1200 data release. A total of 783 subjects from the HCP 1200 data release were used for this analysis (383 males and 400 females). 180 subjects were 22–25 year-old, 343 subjects were 26–30 year-old, 252 subjects were 31–35 year-old, and 8 subjects were above 36 year-old. The HCP 1200 data release had 1096 subjects in total. The Pearson correlation between head motion and the cognitive scores is *r* = −0.17. In other words, some subjects with low cognitive scores had high head motion. While establishing the association between information flow and cognitive scores, we sought to remove confounding effect from head motion. We excluded 211 subjects with significantly high head motion and low cognitive scores during the scans. Specifically, we excluded subject *i*, if $\frac{H{M}_{i}}{DCC{S}_{i}}\times {10}^{3} > 1$, where *HM*_*i*_ is the frame-to-frame head motion estimate (averaged across both day 1 rest runs; HCP: Movement_RelativeRMS_mean) and *DCCS*_*i*_ is the DCCS score for subject *i*, respectively. The correlation between head motion and the cognitive scores is *r* = −0.005 after exclusion. The other excluded subjects had missing time points. The data were acquired in two separate sessions (REST 1 and REST 2) on two different days. Each session contained data from both the left-right (LR) and right-left (RL) phase-encoding runs. The data (REST 1 LR, REST 1 RL, REST 2 LR, REST 2 RL) were concatenated to calculate the information flow metrics. All participants provided written informed consent. Subject recruitment procedures and informed consent forms, including consent to share de-identified data, were approved by the Washington University in St. Louis Institutional Review Board (IRB). All experimental procedures were performed under the guidelines of the HCP, which adhered to the relevant IRB processes related to that project; full details on the HCP have been published previously^60^. The datasets analyzed during the current study are available on the HCP page (https://www.humanconnectome.org/study/hcp-young-adult).

### Data acquisition and preprocessing

The functional imaging data (the WU-Minn HCP Phase II data) were acquired at 2 mm isotropic on a 3T Magnetom Skyra Connectom scanner using a 32-channel head coil. Parameters for the functional scans were: TR = 720 ms, TE = 33 ms, echo spacing (spin echo field) = 0.58 ms, FOV = 208 mm × 180 mm, Matrix = 104 × 90 with 72 slices covering the whole brain, multiband factor of 8, and FA = 52°. There were two resting state functional runs for each participant, each lasted 14.39 mins (1200 time points). The structural data included a pair of T1- weighted image, all acquired at 0.7 mm isotropic voxel resolution, plus ancillary scans, for a session duration of ~40 min: TR = 2400 ms, TE = 2.14 ms, TI = 1000ms, voxel size 0.7*0.7*0.7 mm^3^, FOV = 224 mm, FA = 8°.

Data were preprocessed using the standard pipeline implemented in the Statistical Parametric Mapping (SPM12, http://www.fil.ion.ucl.ac.uk/spm/), following the previously published work^61–64^. The procedure included slice-timing correction, realignment, individual structural-functional image coregistration, Montreal Neurological Institute (MNI) template normalization and spatial smoothing. Additional noise corrections were applied to be consistent with previous work^3^, including removal of linear components related to the 12 motion parameters (six motion parameters plus their first derivatives), regression of mean time courses of the white matter, cerebrospinal fluid and global signal, removal of the linear trend, and low-pass filtering (<0.12 Hz). Secondary data analysis, including calculating information flow metrics, feature selection, and model building, were conducted using the R software. For each subject, we obtained a 268 × 268 asymmetrical information flow metric matrix.

### A three-step procedure for identifying information flow

#### Preamble: Granger-Geweke causality analysis

A random variable X *Granger causes* another variable Y, if the prediction of Y is improved using information of its own past and the past of X, compared with when using only the past information of Y. Formally, let {X_t_}_t∈Z_ be a process, where Z denotes the set of integers each of which corresponds to a time point, and ${{\rm{X}}}_{{\rm{t}}}={({{\rm{X}}}_{{\rm{t}}1},\cdots ,{{\rm{X}}}_{{\rm{t}}{\rm{d}}})}^{{\rm{T}}}$ . For $j\ne k\in \{1,\,\cdots \,{\rm{d}}\}$, ${\{{{\rm{X}}}_{{\rm{tk}}}\}}_{{\rm{t}}\in {Z}_{+}}$ *Granger causes* ${\{{{\rm{X}}}_{{\rm{tj}}}\}}_{{\rm{t}}\in {Z}_{+}}$ if and only if there exists a measurable set A such that

$${P(X}_{{\rm{t}}+1,{\rm{j}}}\in {\rm{A}}|{\{{{\rm{X}}}_{{\rm{s}}}\}}_{{\rm{s}}\le {\rm{t}}})\ne {P(X}_{{\rm{t}}+1,{\rm{j}}}\in {\rm{A}}|{\{{{\rm{X}}}_{{\rm{s}},{\rm{\setminus }}{\rm{k}}}\}}_{{\rm{s}}\le {\rm{t}}})$$

for all t ∈ *Z*_+_, where *Z*_+_ denotes positive integers and {X_s,\k_} is the subvector obtained by removing X_sk_ from X_s_.

Given that the processes are stationary, Granger causality can be implemented via autoregressive (AR) modelling. Formally, consider the following AR processes:

$${{\rm{X}}}_{{\rm{t}}}=\sum _{{\rm{j}}=1}^{{\rm{\infty }}}{\alpha }_{{\rm{j}}}{{\rm{X}}}_{{\rm{t}}-{\rm{j}}}+{\varepsilon }_{{\rm{j}}},\,{{\rm{V}}{\rm{a}}{\rm{r}}(\varepsilon }_{{\rm{j}}})={{\rm{\Sigma }}}_{1}$$

$${{\rm{Y}}}_{{\rm{t}}}=\,\sum _{{\rm{j}}=1}^{\infty }{{\rm{\beta }}}_{{\rm{j}}}{{\rm{Y}}}_{{\rm{t}}-{\rm{j}}}+{{\rm{\eta }}}_{{\rm{j}}},\,{\rm{Var}}({{\rm{\eta }}}_{{\rm{j}}})={{\rm{T}}}_{1}$$

To inquire into the potential predictability of X_t_ on Y_t_, and *vice versa*, we further define

$${{\rm{X}}}_{{\rm{t}}}=\sum _{{\rm{j}}=1}^{\infty }{{\rm{a}}}_{{\rm{j}}}{{\rm{X}}}_{{\rm{t}}-{\rm{j}}}+\sum _{{\rm{j}}=1}^{\infty }{{\rm{b}}}_{{\rm{j}}}{{\rm{Y}}}_{{\rm{t}}-{\rm{j}}}+{{\rm{e}}}_{{\rm{j}}},\,{\mathrm{Var}(e}_{{\rm{j}}})={{\rm{\Sigma }}}_{2}$$

$${{\rm{Y}}}_{{\rm{t}}}=\,\sum _{{\rm{j}}=1}^{\infty }{{\rm{c}}}_{{\rm{j}}}{{\rm{X}}}_{{\rm{t}}-{\rm{j}}}+\sum _{{\rm{j}}=1}^{\infty }{{\rm{d}}}_{{\rm{j}}}{{\rm{Y}}}_{{\rm{t}}-{\rm{j}}}+{{\rm{z}}}_{{\rm{j}}},\,{\mathrm{Var}(z}_{{\rm{j}}})={{\rm{T}}}_{2}$$

where the covariance between the noises is ${\rm{Cov}}({{\rm{e}}}_{{\rm{j}}},\,{{\rm{z}}}_{{\rm{j}}})\,:\,={{\rm{\gamma }}}_{2}$.

Under this framework, the Geweke test^18^ provides a linear-feedback measure. Denote F_X→Y_, F_Y→X_, and ${{\rm{F}}}_{{\rm{X}}\to {\rm{Y}}}$ as the feedback measure from X to Y, from Y to X, and the instantaneous feedback measure between X and Y, respectively. Formally, they can be written as:

$$\begin{array}{rcl}{{\rm{F}}}_{{\rm{Y}}\to {\rm{X}}} & = & \mathrm{ln}(\frac{|{{\rm{\Sigma }}}_{1}|}{|{{\rm{\Sigma }}}_{2}|})\\ {{\rm{F}}}_{{\rm{X}}\to {\rm{Y}}} & = & \mathrm{ln}(\frac{|{{\rm{T}}}_{1}|}{|{{\rm{T}}}_{2}|})\end{array}$$

where |·| denoted matrix determinant.

**Step 1: Granger-Geweke test and Individual-level information flow map**. The information flow varied between different subjects and across brain regions. To quantify the subject-specific information flow, we introduced the node-wise optimal lag Granger-Geweke test to obtain the whole brain information flow metrics. For two corresponding time courses corresponding to a node pair (*i*, *j*) we conducted the Granger-Geweke test to uncover the information feedback between them: *F*_i→j_ and *F*_j→i_. The choice of lag *l*, where x_t−l_ was the lag *l* value of x_t_, was critical to detect the Granger causality between two time courses. Here, we define an optimal choice of lag *l* as one that not only explains a reasonable amount of variance of the data, but also is easy to estimate for large-scale data, using the node-wise Akaike information criterion (AIC)^22^. Formally, we define the optimal lag length between two time courses from nodes *i* to *j*, for a specific subject *k*, as

$${{\rm{l}}}_{{\rm{i}}\to {\rm{j}},\,{\rm{k}}}^{{\rm{opt}}}={{\rm{\min }}}_{{\rm{l}}}\{{{\rm{\alpha }}}_{{\rm{i}}\to {\rm{j}},\,{\rm{k}}}^{{\rm{l}}}\}$$

where ${{\rm{\alpha }}}_{{\rm{i}}\to {\rm{j}}}^{{\rm{l}}}$ is the AIC score of two times courses for nodes *i* to *j* with lag *l*.

**Step 2: Edge selection**. The subject-level information flow maps share common patterns (Fig. 1 (a)), indicating that there are consistent directed edges (*F*-values) in a population. To uncover these common information flow paths, we further investigated information flow in a group. We conducted a node-wise feature selection as follows.

We select an edge from *i* to *j* (namely we select *F*_i→j_), if *ξ*_i→j_ ≤ α, where *ξ*_i→j_ is a feature calculated as the average of the *p*-values across all subjects (1 ≤ *k* ≤ *N*) resulting from the Granger-Geweke tests regarding two time courses obtained from nodes *i* and *j*, namely, ${\xi }_{{\rm{i}}\to {\rm{j}}}=\frac{{\sum }_{{\rm{k}}=1}^{{\rm{N}}}{{\rm{p}}}_{{\rm{i}}\to {\rm{j}}}^{{\rm{k}}}}{{\rm{N}}}$, and α is the feature selection rejection threshold (*e*.*g*. 0.05). Particularly, the feature selection was conducted based on ${p}_{{\rm{i}}\to {\rm{j}}}^{{\rm{k}}}$, for every subject *k*, and for every pair of nodes (*i*, *j*). Specifically, ${p}_{{\rm{i}}\to {\rm{j}}}^{{\rm{k}}}$ is the *p*-value from a Granger-Geweke analysis between two time courses from region *i* to *j*, for subject *k*, under the optimal lag length ${{\rm{l}}}_{{\rm{i}}\to {\rm{j}},{\rm{k}}}^{{\rm{opt}}}$ for the same subject. The resulting directed (significant) edges were information flow paths that were likely to be present across different subjects. They were candidate features used to predict cognition.

**Step 3: Prediction of executive functioning**. Next, we examined if their variability was associated with variability in subject-specific cognition. In the HCP protocol, executive functioning was evaluated by the Dimensional Change Card Sort (DCCS) test. Subjects were asked to sort a series of bivalent test cards, first according to one dimension (*e*.*g*. color), and then according to the other (*e*.*g*. shape).

During the training state, we used leave-one-subject-out cross-validation (LOOCV) in a sample of 550 subjects (70% of the total samples) to further prune edges extracted from Step 2 above. During each iteration, we first conducted feature selection on edges extracted from 549 subjects, and built a regression model. Afterwards, we validated the model using data from the holdout subject. This was iterated 550 times, where each subject was predicted once. Specifically for the feature selection during LOOCV, we correlated the *F*-values from each edge with the DCCS scores in the training set. We chose the edges whose *F*-values had a significant Pearson correlation with the DCCS scores as neurological signatures. Selected edges were subsequently separated to two groups: ones with a positive correlation (positive signatures, or *F*^+^ edges), and ones with a negative correlation (negative signatures, or *F*^−^ edges). As a result of the model development, we built two regression models using the *F*^+^ and *F*^−^ edges in the training data, as follows:

$${\hat{{\rm{y}}}}^{+}={\hat{{\rm{\beta }}}}_{0}^{+}+{\hat{{\rm{\beta }}}}_{1}^{+}\sum _{{\rm{i}}=1}^{{\rm{q}}}\sum _{{\rm{j}}=1}^{{\rm{q}}}{F}_{{\rm{i}}\to {\rm{j}}}^{+}{\hat{{\rm{I}}}}_{{\rm{i}}\to {\rm{j}}}^{+}$$

$${\hat{{\rm{y}}}}^{-}={\hat{{\rm{\beta }}}}_{0}^{-}+{\hat{{\rm{\beta }}}}_{1}^{-}\sum _{{\rm{i}}=1}^{{\rm{q}}}\sum _{{\rm{j}}=1}^{{\rm{q}}}{F}_{{\rm{i}}\to {\rm{j}}}^{-}{\hat{{\rm{I}}}}_{{\rm{i}}\to {\rm{j}}}^{+}$$

where ${F}_{{\rm{i}}\to {\rm{j}}}^{+}$ and ${F}_{{\rm{i}}\to {\rm{j}}}^{-}$ are observed information flow metrics from node *i* to node *j*. ${\hat{{\rm{I}}}}_{{\rm{i}}\to {\rm{j}}}^{+}$ is an indicator function, where ${\hat{{\rm{I}}}}_{{\rm{i}}\to {\rm{j}}}^{+}=1$ if ${F}_{{\rm{i}}\to {\rm{j}}}^{+}$ is associated with a significant information flow, and 0 otherwise. ${\hat{{\rm{I}}}}_{{\rm{i}}\to {\rm{j}}}^{-}$ is similarly defined. ${\hat{{\rm{\beta }}}}_{0}^{+}$, ${\hat{{\rm{\beta }}}}_{0}^{-}$, ${\hat{{\rm{\beta }}}}_{1}^{+}$, and ${\hat{{\rm{\beta }}}}_{1}^{-}$ are estimated regression weights. *q* is the number of nodes. Finally, ${\hat{{\rm{y}}}}^{+}$ and ${\hat{{\rm{y}}}}^{-}$ are predicted DCCS scores using each model.

To conduct out-of-sample predication, we first computed the *F*-values (${F}_{{\rm{i}}\to {\rm{j}}}^{+}$ and ${F}_{{\rm{i}}\to {\rm{j}}}^{-}$) for each individual in the testing data consisting of 233 novel subjects (30% of the total samples). We then integrate the parameter (${\hat{{\rm{\beta }}}}_{0}^{+}$, ${\hat{{\rm{\beta }}}}_{0}^{-}$, ${\hat{{\rm{\beta }}}}_{1}^{+}$, ${\hat{{\rm{\beta }}}}_{1}^{-}$, ${\hat{{\rm{I}}}}_{{\rm{i}}\to {\rm{j}}}^{+}$, and ${\hat{{\rm{I}}}}_{{\rm{i}}\to {\rm{j}}}^{-}$) estimated from the training data with the *F*-values from the testing data to predict DCCS scores (${\hat{{\rm{y}}}}^{+}$ and ${\hat{{\rm{y}}}}^{-}$) for the novel subjects. The prediction was done without any further modeling of the testing data. Finally, we accessed the efficacy and power of the model by correlating the predicated and observed DCCS scores in the training data.

## Supplementary information

Supplementary Information
