# Supplementary material for: Resting-state brain information flow predicts cognitive flexibility in humans
Source: Sci Rep. 2019 Mar 7;9:3879. doi: 10.1038/s41598-019-40345-8 (PMC6406001; doi:10.1038/s41598-019-40345-8)
Supplement: Supplementary file 1 — Supplementary Information [file 41598_2019_40345_MOESM1_ESM.pdf]

Supplementary Materials

Resting-state brain information flow predicts cognitive flexibility in humans

Oliver Y. Chén, Hengyi Cao, Jenna M. Reinen, Tianchen Qian,

Jiangtao Gou, Huy Phan, Maarten De Vos, Tyrone D. Cannon

Remarks on edge selection in behavior prediction

The edge selection was performed via a two-step feature selection procedure (see **Fig. 1** in the texts). During the first step we selected an edge, if its average *p-value* (obtained from the GCA) across all subjects was smaller than a threshold. At the threshold of $p < 0.1$ this selected a total of 13,022 directed edges (as a comparison, at the threshold of $p < 0.05$, this selected 3,927 edges; see the next section for a comparison using different thresholds). The second step edge selection was conducted within a predictive modeling framework (see **Fig. 1** and **Method** in the texts for more details).

Notably, the selected edges with large *F*-values across subjects were from the cerebral cortex to bilateral prefrontal ($\mu_F \geq 6.60$), parietal ($\mu_F \geq 8.30$), temporal ($\mu_F \geq 8.11$) and motor regions ($\mu_F \geq 9.38$), as well as flow within the temporal region ($\mu_F \geq 10.37$) (see **Fig. 7** in the texts).

At the network level, within-network information flow was strong in the majority of the examined networks, in particular the medial frontal ($F \geq 7.24$), frontoparietal ($F \geq 7.59$), the default-mode ($F \geq 6.60$), and V1 ($F \geq 18.90$). Relatively large between-network information flow was also observed between the higher-order functional networks that are most pertinent to cognitive functions including the medial frontal, frontoparietal and the default-mode networks ($F \geq 6.60$) (see **Fig. 2** in the texts).

Interpretation of *F*-values from a Granger-Geweke analysis as information flows in the brain

We used *F*-values in the sense where a large $F_{Y \to X}$ value suggests that past information from region Y can improve the (explanation of) current neural activation of region X.

The reason that *F*-value is a reasonable metric for quantifying the directed connectivity between two regions can be interpreted in the context of Granger-Geweke causality, from which each *F*-value is derived. Specifically, consider the following two autoregressive (AR) processes obtained from two time series from two brain areas X and Y:

$$X_t = \sum_{j=1}^{\infty} \alpha_j X_{t-j} + \varepsilon_j, \quad \mathrm{Var}(\varepsilon_j) = \Sigma_1$$

$$X_t = \sum_{j=1}^{\infty} a_j X_{t-j} + \sum_{j=1}^{\infty} b_j Y_{t-j} + e_j, \quad Var(e_j) = \Sigma_2$$

The first AR model is aiming at predicting the activity of region X at time t (X_t) using all the past information of X, and Σ_1 is the variability of X_t that cannot be explained by past information of X. The second AR model further adds in the past information of Y to predict X_t; namely, it uses both X's past information and Y's past information to predict X_t, and Σ_2 is the variability of X_t that cannot be explained by past information of both X and Y. If brain area Y feeds information into X, then the past information of Y must arrive at X after some time – this could be happening continuously. If the past information of Y can improved the explaining of X_t, then Σ_2 must be smaller than Σ_1 (in other words, adding past information of Y into the first AR model reduces the information not explained, or Σ_1).

The Granger-Geweke *F*-value from region Y to X is defined as $F_{Y\rightarrow X} = \ln\frac{|\Sigma_1|}{|\Sigma_2|}$. Quantitatively speaking, it is the log of the ratio of the residual variances before and after adjusting for Y. Note that since $\ln\frac{|\Sigma_1|}{|\Sigma_2|}$ can be written as $\ln|\Sigma_1| - \ln|\Sigma_2|$, $F_{Y\rightarrow X}$ therefore measures the difference between the information (in terms of varibility) not explained in the first AR model and the information not explained in the second AR model. In simpler terms, it quantifies the reduction of information-not-explained (at the log-scale) from one brain area when additional past information of another brain area is included. When $F_{Y\rightarrow X}$ is very large, it means that Σ_2 is much smaller than Σ_1, namely adding past information of Y improves the prediction of current status of X (X_t).

Optimal lags associated with *F*-values

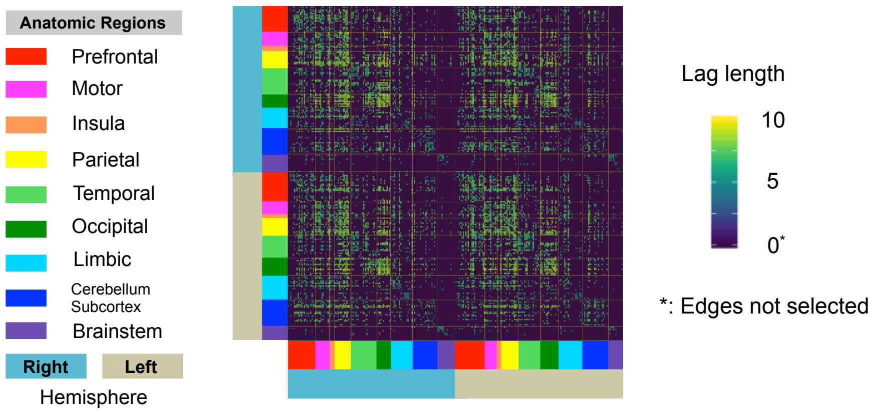

Supplementary Figure 1: The optimal lag length corresponding to each edge is arranged according to their anatomic regions. The brighter the color is, the larger the edge optimal lag is. Those with 0 entries correspond to edges that were not selected. In general, the cerebrum was associated with relatively larger optimal time lag, where the subcortex and cerebellum were associated with relatively smaller lag.

The results showed that lager optimal lags were primarily present in the prefrontal, motor, parietal, temporal, and occipital cortices, where the cerebellum, subcortex, and brain stem were associated with relatively small optimal time lag. This finding may suggest that the information processing and transfer at subcortical and cerebellum are relatively faster than those at the cerebrum.

Left-right and right-left phase encoding in the Human Connectome Project (HCP) Data

Unlike the common EPI phase encoding strategy that is applied in the anterior–posterior (A–P) or posterior–anterior (P–A) direction, the HCP data used a “left–right and right–left” direction strategy. The aim of this strategy was to minimize the field of view (FoV) and the number of lines of k-space during phase encoding and therefore reduce the distortion and blurring[1].

Comparison of signal-to-noise ratio (SNR) between subcortical regions and the whole brain

To examine whether subcortical regions are associated with lower SNR, we computed voxel-wise SNR maps for each individual[2]. In particular, the SNR for a given voxel is defined as the mean of that voxel across all time points over the standard deviation of that voxel. We computed the SNRs for four subcortical nodes (*i.e.* bilateral caudate and bilateral putamen) and compared the derived SNRs with that from the whole brain. We found that, for all of the four regions, the SNRs were significantly higher than that from the whole brain ($p < 0.001$), suggesting that subcortical regions are not associated with poor signal, at least in the sample we used.

References:

1. Smith, S. M. *et al.* Resting-state fMRI in the Human Connectome Project. *Neuroimage* **80,** 144–168 (2013).
2. Murphy, K., Bodurka, J. & Bandettini, P. A. How long to scan? The relationship between fMRI temporal signal to noise ratio and necessary scan duration. *Neuroimage* **34,** 565–574 (2007).
